# Attentional Symptom Expression Moderates the Role of Vocabulary in Reading Comprehension Among Fifth-Grade Students

**DOI:** 10.3390/jintelligence14050080

**Published:** 2026-05-06

**Authors:** Vered Markovich, Tami Katzir, Shoshi Dorfberger

**Affiliations:** 1 Edmond J. Safra Brain Research Center for the Study of Learning Disabilities, University of Haifa, Haifa 3103301, Israel; katzirta@gmail.com; 2 Department of Special Education, Gordon Academic College of Education, Haifa 3570503, Israel; shoshid@gordon.ac.il

**Keywords:** reading comprehension, attentional variability, cognitive-linguistic skills

## Abstract

Previous research has linked inattentive symptom expression to reading difficulties, primarily in clinically referred samples or using indirect academic indicators. The present study examines attentional variability in a nonreferred community-based school sample with no clinical diagnoses or referrals, using standardized reading measures. The study further tested whether attentional variability moderates associations between cognitive linguistic skills and reading comprehension. 267 fifth grade students (mean age = 10.8 years) completed standardized measures of word reading efficiency, vocabulary knowledge, working memory, and reading comprehension. Based on parent and teacher ratings, approximately one-third of the sample showed elevated levels of inattentive symptom expression. Regression analyses indicated that vocabulary knowledge and word reading efficiency accounted for the largest proportion of explained variance in reading comprehension, whereas working memory showed weaker associations. Moderation analyses revealed that attentional symptom expression selectively moderated the relationship between vocabulary knowledge and reading comprehension, such that the positive association between vocabulary and comprehension was attenuated among students with elevated attentional symptom expression. No moderation effects were observed for word reading efficiency or working memory. These findings suggest that attentional variability is differentially related to specific cognitive linguistic components involved in reading comprehension.

## 1. Introduction

Reading comprehension depends on the coordinated use of multiple cognitive-linguistic skills, including word reading efficiency, vocabulary knowledge, and working memory. Individual differences in how effectively these skills are deployed during reading are associated with substantial variability in comprehension outcomes (Kieffer & Christodoulou, 2020; Perfetti & Stafura, 2014; Peng et al., 2018). Extensive cross-linguistic evidence shows that decoding plays a stronger role in early reading, whereas language skills increasingly account for variability as readers develop fluency (García & Cain, 2014; Tighe & Schatschneider, 2014). However, although this framework specifies which component skills are required, it provides a limited explanation for why students with comparable decoding and language skills often differ substantially in comprehension performance. Increasing attention has therefore been directed toward domain-general regulatory processes that influence how these skills are coordinated and applied during reading (Diamond, 2013; Follmer, 2018).

Contemporary frameworks conceptualize comprehension as the joint outcome of linguistic knowledge and regulatory processes that support attention, integration, and monitoring. In particular, the Direct and Indirect Effects Model of Reading (DIER) proposes that attentional and executive skills are often conceptualized as influencing comprehension indirectly by supporting the acquisition and coordinated use of linguistic knowledge (Kim, 2020). From this perspective, comprehension reflects not only what readers know, but also how efficiently they apply that knowledge during ongoing reading.

Not all reading-related skills place the same demands on regulation. Some skills operate relatively automatically, whereas others require sustained attention and active monitoring to be used effectively. Vocabulary knowledge supports semantic access, inference generation, and meaning integration (Perfetti & Stafura, 2014). Its effective use during comprehension depends on maintaining focus and monitoring coherence. In contrast, word reading efficiency relies more heavily on automatized decoding processes and typically imposes fewer attentional demands (Kieffer & Christodoulou, 2020). Working memory supports the temporary maintenance and manipulation of textual information and contributes additively to comprehension performance (Peng et al., 2018). Together, this suggests that attentional and regulatory constraints may affect specific component skills differently rather than influencing comprehension uniformly (Morris & Lonigan, 2022).

Recent meta-analytic evidence further clarifies the relative contribution of cognitive and linguistic components. Across nearly two hundred studies, language-based skills such as vocabulary and listening comprehension show the strongest and most proximal associations with reading outcomes, whereas executive functions, including working memory, demonstrate smaller and largely indirect effects that are substantially reduced once linguistic skills are controlled (Peng et al., 2018, 2022). These findings suggest that comprehension is primarily supported by language knowledge, while domain-general regulation influences performance through the efficiency with which such knowledge is deployed. Consequently, regulatory constraints are expected to differentially affect specific cognitive-linguistic skills, particularly those that rely on sustained semantic integration and controlled processing, such as vocabulary-dependent comprehension (Perfetti & Stafura, 2014).

This regulatory perspective is especially relevant in the context of attentional variability. Inattentive behaviors have repeatedly been associated with lower academic achievement and weaker reading performance (Friedman et al., 2017; Martinussen & Mackenzie, 2015; Miller et al., 2013). However, much of this evidence derives from clinically referred samples or from studies relying on parent or teacher-reported academic indicators rather than standardized reading assessments. For example, inattentive symptoms predicted poorer academic functioning even in nonreferred school populations, although outcomes were often indexed through ratings rather than direct performance measures (Öner et al., 2019; Swanson et al., 2018). As a result, less is known about how naturally occurring attentional variability constrains objectively measured cognitive-linguistic skills that support reading comprehension. To address this gap, the present study examines attentional variability in a nonreferred sample. This sample is a community-based cohort of fifth-grade students. None had a formal diagnosis of specific learning or attention disorders, and none had been referred for evaluation or services by fifth grade. The cohort was followed from kindergarten. The sample therefore reflects naturally occurring variability in attentional symptom expression based on parent and teacher reports. Importantly, in school-based research, such variability is typically assessed dimensionally through parent and teacher report questionnaires aligned with DSM-5 criteria for inattentive symptom expression rather than through formal clinical diagnoses (American Psychiatric Association, 2022), allowing examination of attentional regulation across the full range of functioning within typical classroom populations.

Hebrew is a relatively transparent orthography characterized by high grapheme–phoneme consistency (Frost et al., 1987; Share, 2008; Share & Bar-On, 2018). Decoding demands are typically reduced once basic reading skills are acquired, especially by upper elementary school. By fifth grade, most instructional materials are presented with partial or no vowelization (niqqud), shifting the emphasis from phonological decoding to lexical access, morphological processing, and contextual integration (Katzir et al., 2012; Shany et al., 2012). Moreover, the root-and-pattern morphological structure of Hebrew places increased demands on working memory and attentional regulation, as readers must actively maintain, integrate, and disambiguate morphologically related word forms during comprehension (Frost, 2012; Haddad et al., 2018).

Fifth grade represents a transition from decoding-focused to comprehension-focused reading, characterized by increasing text complexity and heightened demands on attentional regulation and monitoring (Katzir et al., 2012; Roebers, 2017). At this stage, vocabulary knowledge expands rapidly, whereas regulatory systems that support sustained attention and monitoring are still consolidating. In this context, decoding becomes largely automatized by upper elementary school, and comprehension increasingly depends on higher-order linguistic integration and regulatory processes. By fifth grade, students transition from “learning to read” to “reading to learn”, and variability in regulatory and linguistic skills becomes increasingly central to comprehension performance. This developmental stage therefore provides an opportunity to evaluate attentional constraints beyond basic word recognition. Examining comprehension at this stage may clarify how attentional and regulatory differences shape the functional contribution of component skills beyond basic decoding demands.

Although prior research has documented associations between ADHD symptomatology and reading outcomes, most studies have focused on clinically referred samples or on mean-level group differences (e.g., Avramovich & Yeari, 2022; Martinussen & Mackenzie, 2015; Miller et al., 2013; Swanson et al., 2018). Less is known about how variability in inattentive symptom expression within community samples influences the strength of relations between component skills and comprehension. A moderation framework may directly address this question by testing whether associations between predictors and outcomes differ as a function of attentional variability.

The present study examined whether inattentive symptom expression, assessed through parent and teacher rating scales, moderates the relations between word reading efficiency, vocabulary knowledge, working memory, and reading comprehension in a nonreferred sample of fifth-grade students. Rather than comparing groups, we tested whether attentional variability changes the magnitude of associations between these cognitive-linguistic skills and comprehension. Based on regulatory accounts of skill deployment (Kim, 2020), vocabulary was expected to be particularly sensitive to attentional variability because its effective use depends on sustained semantic integration and monitoring.

**Figure 1:**
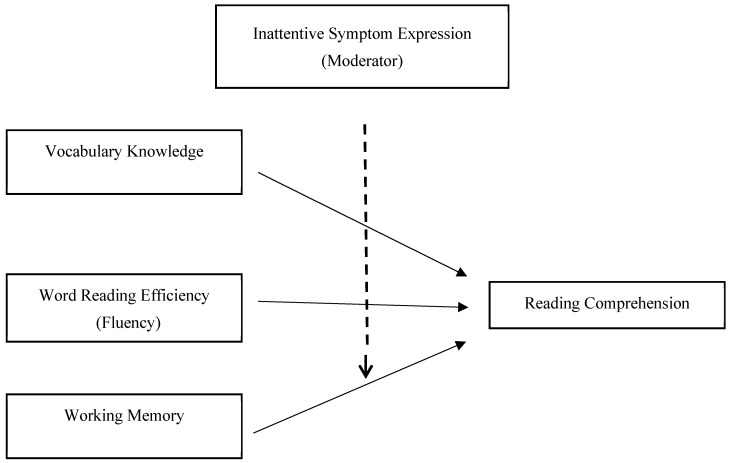
Conceptual model illustrating inattentive symptom expression as a moderating factor in the relations between cognitive-linguistic components and reading comprehension.

Figure 1 presents the conceptual model tested in the present study, illustrating inattentive symptom expression as a moderating factor in the relations between cognitive-linguistic components and reading comprehension.

Based on this framework, the following hypotheses were tested:

**H1.**  *Students with elevated inattentive symptom expression were expected to show lower performance on cognitive-linguistic skills*.

**H2.**  *Word reading efficiency, vocabulary knowledge, and working memory were expected to be positively associated with reading comprehension, with vocabulary knowledge expected to account for the largest share of variance, followed by word reading efficiency, and working memory showing the weakest contribution*.

**H3.**  *Attentional variability would attenuate the vocabulary–comprehension association more strongly than associations involving word-reading efficiency or working memory*.

## 2. Materials and Methods

### 2.1. Participants

The study included 267 fifth-grade students (mean age = 10.8 years, SD = 0.45), approximately evenly distributed between boys (48.7%) and girls (51.3%), recruited from elementary schools in Israel. All participants were native Hebrew speakers. Hebrew was the primary language of instruction and assessment for all participants, and none were in the process of learning Hebrew as a new language at the time of assessment. Inclusion criteria required no diagnosed specific learning or attention disorders, neurological conditions, or sensory impairments, based on school records, and no prior referral for clinical evaluation or special education services. The sample was drawn from an ongoing longitudinal cohort followed from kindergarten. Testing sessions were conducted individually in a quiet environment, with each session lasting approximately 45–60 min. Standardized instructions were provided for all tasks, and breaks were allowed as needed to reduce fatigue.

### 2.2. Materials and Measures

#### 2.2.1. Reading Comprehension

Reading comprehension was assessed using the Developmental TAMAR computerized measure. See description in previous Hebrew reading studies (e.g., Kilim & Prior, 2025). The task required participants to read two short texts (150 words each) and respond to five multiple-choice questions per passage under a ten-minute constraint. To minimize memory demands, texts remained available during response selection. Reported reliability (α = 0.76–0.84).

#### 2.2.2. Vocabulary Knowledge

Vocabulary knowledge was measured using the Elul Test (Shatil et al., 2007). The task includes multiple-choice items requiring the selection of a pictorial representation corresponding to a target word. Scores reflect the total number of correct responses (range 0–35). The test is widely used with Hebrew-speaking elementary school populations and demonstrates good reliability (α = 0.86).

#### 2.2.3. Reading Fluency

Reading fluency was assessed using a Hebrew adaptation of the test of Word Reading Efficiency (TOWRE; Torgesen et al., 1999). Participants read real words and pseudowords within a 45 s time limit. Performance was indexed by the number of correctly read items. Previous studies report high reliability for this measure (α = 0.89–0.94).

#### 2.2.4. Working Memory

Working memory was measured using the Digit Span subtest from the WISC-III (Wechsler, 1991), a commonly used index of verbal working memory in developmental research, including Hebrew-speaking populations (e.g., Raz & Shaul, 2025; Tal et al., 2025). Across successive Wechsler versions, Digit Span has shown stable construct validity and is commonly used as an index of verbal working memory capacity (Egeland et al., 2026). The task included forward and backward spans, and a composite raw score was computed. Reliability was acceptable (α = 0.82). In the present study, this measure was included as an exploratory control variable.

#### 2.2.5. Assessment of Attentional Symptom Expression

Attentional variability was assessed using a DSM-5–based rating questionnaire adapted for research use with Hebrew-speaking children. The questionnaire includes 18 items reflecting inattentive and hyperactive/impulsive behaviors, consistent with the symptom domains described in the Diagnostic and Statistical Manual of Mental Disorders (DSM-5; American Psychiatric Association, 2022). In accordance with DSM-5 criteria for children, symptoms are considered indicative of elevated Inattentive symptom expression when at least six symptoms of inattention and/or hyperactivity/impulsivity are endorsed, persist for a minimum of six months, appear before the age of 12, and are evident in two or more settings.

Inattentive symptom expression refers to observable difficulties in sustaining attention, including frequent distraction, incomplete task execution, difficulty following instructions, and reduced persistence during cognitively demanding activities. In classroom contexts, these behaviors may manifest as inconsistent engagement during reading, skipping portions of text, or difficulty maintaining attention across extended passages. Hyperactive/impulsive symptom expression reflects excessive motor activity, impulsive responding, and reduced inhibitory control, and may be expressed as restlessness, interrupting, responding prematurely, and difficulty waiting for turns. During reading tasks, such behaviors may result in rushed responding, reduced monitoring, and premature disengagement from the text (American Psychiatric Association, 2022; Cole et al., 2023; Parks et al., 2022; Swanson et al., 2018).

Ratings were completed independently by both parents and teachers in order to obtain a multi-informant perspective across home and school contexts. Multi-informant assessment is commonly used in educational and developmental research to capture variability in attentional functioning across settings.

In the present study, questionnaire scores were used to index levels of Inattentive symptom expression rather than to establish a clinical diagnosis. Children were identified as exhibiting elevated inattentive symptom expression when ratings exceeded established symptom thresholds on parent and/or teacher reports, consistent with prior non-clinical research examining attentional variability in school-age samples (e.g., Ramos et al., 2020; Swanson et al., 2018). This approach allowed examination of individual differences in attentional regulation within a nonreferred educational sample, aligning with the study’s focus on attentional variability rather than categorical diagnosis.

Previous research has demonstrated high internal consistency for the Hebrew version of this questionnaire. For teacher reports, reliability coefficients were α = 0.93 overall (α = 0.91 for hyperactivity/impulsivity; α = 0.89 for inattention). For parent reports, overall reliability was α = 0.90 (α = 0.81 for hyperactivity/impulsivity; α = 0.89 for inattention).

### 2.3. Statistical Analysis

Sample size adequacy was evaluated using a post hoc power analysis conducted with GPower 3.1.9.7. For linear regression analyses with 5 predictors, assuming f^2^ = 0.15 and α = 0.05, the sample size (N = 267) yielded an achieved power of 0.99 (Cohen, 1988; Faul et al., 2009). Data analysis was conducted using IBM SPSS 29, following a structured analytic approach. Intraclass correlation coefficients (ICCs) were examined to assess potential classroom-level clustering effects. All ICC values were below 0.05, indicating minimal between-class variance. Accordingly, multilevel modeling was not warranted, and all analyses were conducted at the individual level.

Descriptive statistics were computed for all study variables, including means, standard deviations, and confidence intervals. Distributional assumptions were examined using the Kolmogorov–Smirnov and Shapiro–Wilk tests. Normality was assessed using the Kolmogorov–Smirnov and Shapiro–Wilk tests. Pearson correlation analyses were conducted to explore bivariate associations among vocabulary knowledge, word reading efficiency, working memory, and reading comprehension.

Hierarchical multiple regression analyses with blockwise entry were then conducted to examine the proportion of variance in reading comprehension associated with each cognitive-linguistic component. Gender and attentional symptom expression were entered as control variables in the first step, followed by vocabulary knowledge, word reading efficiency, and working memory in subsequent steps. Prior to model estimation, assumptions of linearity, normality, homoscedasticity, and multicollinearity were examined and met.

To examine whether attentional symptom expression was associated with differences in the strength of relations between cognitive-linguistic components and reading comprehension, moderation analyses were conducted using the PROCESS macro Model 1 (Hayes, 2018). Separate models were estimated for vocabulary knowledge, word reading efficiency, and working memory. In each model, reading comprehension served as the dependent variable, the cognitive-linguistic measure as the independent variable, and attentional symptom expression as the moderator. Gender was included as a covariate to account for its established association with attentional symptom expression. Interaction effects were examined, and conditional effects were probed at different levels of attentional symptom expression.

## 3. Results

### 3.1. Sample Characteristics

Of the 267 fifth-grade students who participated in the study, 84 students (31.5%) exhibited elevated levels of Inattentive symptom expression based on parent and/or teacher ratings. Symptom expression was identified using multi-informant questionnaire data obtained from parents and teachers. Students were classified as exhibiting elevated symptom expression if at least one informant reported symptom frequencies exceeding the established threshold. These classifications were used solely for analytic purposes and do not represent clinical diagnoses.

To further contextualize the distribution of inattentive symptom expression, 31.1% of the sample showed no inattentive symptoms, 21.7% showed 1–2 symptoms, 15.7% showed 3–5 symptoms, 17.2% showed 6–7 symptoms, and 14.2% showed 8–9 symptoms, based on the highest rating reported by either parent or teacher.

There were no significant age differences between these two groups. Gender distribution differed significantly between students with elevated Inattentive symptom expression and those without. Among boys (n = 130), 52 students (40.0%) exhibited elevated symptom expression, whereas among girls (n = 137), 32 students (23.7%) met this criterion.

Chi-square test indicated a significant association between gender and Inattentive symptom expression, χ^2^(1) = 8.123, *p* = .004. No significant differences were observed across the school sector (religious vs. secular) or socioeconomic deciles. A detailed description of sample characteristics is presented in Table 1.

### 3.2. Group Differences in Cognitive-Linguistic Measures

To examine Hypothesis 1, independent samples *t*-tests were conducted to compare cognitive-linguistic performance between students with elevated Inattentive symptom expression and those without.

Word reading efficiency differed significantly between groups, t(265) = 4.191, *p* < .001, with a mean difference of 7.28 (95% CI [3.85, 10.71]), indicating improved performance in students without elevated Inattentive symptom expression.

Vocabulary knowledge also differed significantly, t(265) = 3.181, *p* = .002, with a mean difference of 2.82 (95% CI [1.07, 4.57]), suggesting that students without elevated Inattentive symptom expression had significantly larger vocabulary sizes.

Working memory performance was also significantly different between groups, t(265) = 2.515, *p* = .013, with a mean difference of 0.36 (95% CI [0.08, 0.63]), indicating that students without elevated Inattentive symptom expression had significantly higher working memory scores. Descriptive statistics are presented in Table 2.

Given the observed association between gender and Inattentive symptom expression, additional analyses were conducted to examine potential gender differences in word-reading efficiency, vocabulary knowledge, and working memory. No significant gender differences were observed for any of these measures.

### 3.3. Associations Between Cognitive-Linguistic Measures and Reading Comprehension

To examine Hypothesis 2, Pearson correlation analyses were conducted to assess bivariate associations between cognitive-linguistic measures and reading comprehension. The analysis revealed positive and statistically significant correlations between reading comprehension and all predictor variables (see Table 3).

Given these significant associations, a hierarchical multiple regression analysis was conducted to examine the unique variance in reading comprehension associated with each cognitive-linguistic component. Gender and Inattentive symptom expression were included as control variables. The regression model was statistically significant, F(5, 257) = 30.41, *p* < .001, explaining 37.2% of the variance in reading comprehension (R^2^ = 0.372; adjusted R^2^ = 0.360).

Vocabulary knowledge (B = 0.136, β = 0.385, *p* < .001) and word reading efficiency (B = 0.051, β = 0.301, *p* < .001) accounted for the largest proportions of explained variance. These results indicate that an increase in word reading efficiency and vocabulary size is significantly associated with better reading comprehension scores. Working memory showed a smaller but statistically significant association (B = 0.210, β = 0.104, *p* = .047). Neither gender nor Inattentive symptom expression presence was significantly associated with reading comprehension after accounting for cognitive-linguistic variables (see Table 4).

### 3.4. Moderation Analysis

To examine whether Inattentive symptom expression was associated with differences in the strength of relations between cognitive-linguistic components and reading comprehension (Hypothesis 3), three separate moderation analyses were conducted using the PROCESS macro Model 1 (Hayes, 2018).

In each model, reading comprehension served as the dependent variable, one cognitive-linguistic component was entered as the independent variable, Inattentive symptom expression as the moderator, and gender as a covariate. Interaction effects were examined, and conditional effects were probed at different levels of Inattentive symptom expression.

Two of the three models (word reading efficiency and working memory) did not yield significant interaction effects.

In contrast, the model examining vocabulary knowledge revealed a significant interaction effect. The model was statistically significant, F(4, 262) = 26.96, *p* < .001, explaining 29.2% of the variance in reading comprehension (R^2^ = 0.292). The interaction between vocabulary knowledge and Inattentive symptom expression accounted for an additional 1.5% of variance in reading comprehension beyond the main effects (ΔR^2^ = 0.015, F(1, 262) = 5.64, *p* = .018), representing a small but statistically reliable effect. The significant negative moderation effect (B = −0.09, SE = 0.04, t = −2.36, 95% CI [−0.168, −0.016]), indicating that the strength of the association between vocabulary knowledge and reading comprehension becomes weaker as a function of Inattentive symptom expression.

**Figure 2:**
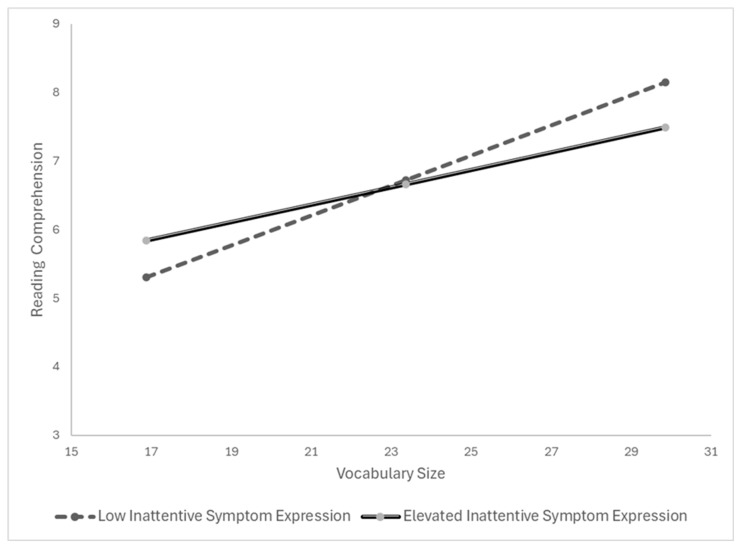
Interaction of Vocabulary and Inattentive Symptom Expression on Reading Comprehension.

To clarify the practical significance of this interaction, we examined the conditional effects of vocabulary knowledge on reading comprehension separately for students with and without elevated Inattentive symptom expression. Among students without elevated Inattentive symptoms, vocabulary knowledge was a strong and significant predictor of reading comprehension (B = 0.22, SE = 0.02, t = 9.17, *p* < .001, 95% CI [0.172, 0.266]). Among students with elevated Inattentive symptoms, the association remained statistically significant but was notably weaker (B = 0.13, SE = 0.03, t = 4.17, *p* < .001, 95% CI [0.067, 0.187]). Conditional effects are presented in Table 5 and illustrated in Figure 2.

## 4. Discussion

The present study examined whether inattentive symptom expression is associated with differences in the functional contribution of cognitive linguistic skills to reading comprehension in fifth grade. Rather than focusing on clinically referred samples or categorical diagnoses, we investigated naturally occurring attentional variability within a nonreferred school population drawn from a community-based cohort. We used standardized reading measures. This design allowed us to evaluate how specific skills relate to comprehension. It also allowed us to test whether these relations vary as a function of inattentive symptom expression.

Three main findings emerged. First, students with elevated inattentive symptom expression demonstrated lower performance across word reading efficiency, vocabulary knowledge, and working memory. Second, all three skills were positively associated with reading comprehension, although their contributions differed. Vocabulary knowledge accounted for the largest share of variance. Word reading efficiency accounted for the next largest shared variance. Working memory showed the smallest contribution. Third, inattentive symptom expression selectively moderated the relation between vocabulary knowledge and reading comprehension. The association was weaker among students with elevated inattentive symptom expression. No moderation effects were observed for word reading efficiency or working memory.

### 4.1. Cognitive–Linguistic Predictors of Reading Comprehension

Vocabulary knowledge emerged as the strongest correlated skill with reading comprehension. This finding aligns with theoretical and meta-analytic evidence showing that language-based skills are the most proximal contributors to comprehension outcomes (Peng et al., 2018, 2022; Perfetti & Stafura, 2014). Vocabulary supports semantic access, inference generation, and integration of ideas across sentences. These processes are central for constructing coherent representations of text. They also require sustained attention and ongoing monitoring. This may increase sensitivity to attentional constraints (Follmer, 2018; Roebers, 2017). The results, therefore, support the view that vocabulary-dependent comprehension relies not only on knowledge. It also relies on the ability to sustain attention during meaning construction.

Word reading efficiency showed a smaller but significant association with reading comprehension. Fluent decoding seems to reduce processing demands and allows readers to allocate resources to higher-level understanding (Katzir et al., 2012; Kieffer & Christodoulou, 2020). Developmental accounts indicate that early reading relies on effortful serial decoding. With practice, decoding becomes increasingly automatized. As decoding becomes more automatic, fewer attentional resources are required. This allows greater focus on comprehension (Balhinez & Shaul, 2024). In transparent orthographies such as Hebrew, this transition tends to occur earlier and more rapidly (Frost et al., 1987; Share, 2008; Share & Bar-On, 2018). By fifth grade, decoding is typically efficient and stable. As word recognition becomes more automatic, its contribution to comprehension may place fewer demands on attentional control. This developmental pattern may help explain the absence of moderation for word-reading efficiency in the current research.

Working memory demonstrated the weakest association with reading comprehension. Working memory was included as an exploratory control variable rather than a primary predictor. Its modest contribution is consistent with research showing that executive functions often explain only limited additional variance once language skills are considered (Kilim & Prior, 2025; Peng et al., 2018; Ramos et al., 2020). In addition, the digit span task primarily reflects storage capacity. It does not directly capture continuous regulation during reading. This distinction may help explain the limited relation between working memory and comprehension observed here. Together, these findings also align with the Simple View of Reading. This framework emphasizes the combined roles of decoding and linguistic comprehension (Gough & Tunmer, 1986). At this stage of development, language knowledge appears to continuously play the dominant role.

### 4.2. Inattentive Symptom Expression as a Moderator

The most distinctive finding concerns the moderation analyses. Inattentive symptom expression was associated with a reduced strength of the relation between vocabulary knowledge and comprehension. Vocabulary predicted comprehension for all students. However, the association was weaker among those with elevated inattentive symptom expression. This pattern suggests that attentional variability may influence how effectively linguistic knowledge is used during reading. Successful use of vocabulary requires sustained focus. It also requires monitoring of coherence and integration of information across sentences. When attention fluctuates, readers may have difficulty maintaining these processes. This may reduce the benefit of their lexical knowledge for comprehension. This interpretation is consistent with evidence linking inattention with weaker academic and reading outcomes (Friedman et al., 2017; Martinussen & Mackenzie, 2015; Miller et al., 2013). At the same time, direct measures of attentional control were not included. Therefore, causal conclusions cannot be drawn.

In contrast, inattentive symptom expression did not moderate relations involving word reading efficiency or working memory. These components may rely less on sustained online attention during comprehension. Word recognition becomes increasingly automatic at this age. Working memory capacity reflects more general constraints. As a result, these skills may be less sensitive to fluctuations in attention during the reading tasks used in the present research.

### 4.3. Implications for Models of Reading

Contemporary models emphasize that reading comprehension reflects both linguistic knowledge and regulatory processes that support attention and monitoring (Diamond, 2013; Duke & Cartwright, 2021; Kim, 2020). The present findings are consistent with this perspective. They suggest that attentional variability may shape how strongly specific linguistic skills contribute to comprehension. They do not suggest a uniform effect across all components. The results also extend prior work in two ways. First, they examine these relations within a nonreferred sample. Second, they test the moderation of functional contributions rather than focusing only on mean level differences.

The Hebrew orthographic context may further clarify these patterns. Because decoding demands are reduced in transparent orthographies, comprehension differences at fifth grade may more clearly reflect higher-order linguistic processes and sustained attention rather than basic word recognition (Frost et al., 1987; Share, 2008; Share & Bar-On, 2018). Within this context, the observed moderation suggests that inattentive symptom expression is related to how effectively cognitive linguistic skills are applied during reading, rather than to limitations in decoding. Cross-linguistic and longitudinal research is needed to evaluate this possibility directly.

### 4.4. Educational Implications

From an educational perspective, the findings suggest that vocabulary instruction alone may not fully address comprehension difficulties among students with elevated inattentive symptom expression. These students may possess adequate lexical knowledge. However, they may experience difficulty sustaining attention during reading. Instruction that combines vocabulary development with structured support for monitoring and comprehension strategies may therefore be beneficial. These implications require further direct testing in intervention studies.

In practical terms, combined vocabulary and attentional support may be implemented through instructional approaches that explicitly scaffold both meaning construction and sustained engagement. For example, teachers may integrate structured vocabulary instruction with guided reading activities that include frequent prompts for monitoring comprehension, maintaining focus, and checking understanding (Perfetti & Stafura, 2014; Duke & Cartwright, 2021). Instructional practices such as segmenting texts into shorter units, providing explicit goals for each reading segment, and embedding brief opportunities for self-monitoring may help sustain attention during meaning integration. In addition, external supports such as visual cues, teacher-guided questioning, and structured discussion can reduce attentional demands while supporting vocabulary-based comprehension processes (Follmer, 2018; Roebers, 2017).

Early identification of inattentive symptom expression through DSM-5–based behavioral screening tools may support more targeted and mechanism-based support, rather than relying solely on language-focused instruction. Multi-informant reports from parents and teachers can provide complementary information about attentional functioning across contexts and may help guide coordinated planning between home and school.

### 4.5. Limitations and Future Directions

Several limitations should be noted. Inattentive symptom expression was assessed using parent and teacher reports rather than clinical diagnosis. The study also did not include direct behavioral measures of attention or executive control. In addition, working memory was assessed using the Digit Span task, which primarily captures verbal storage capacity and provides a well-established index of working memory. However, it does not fully reflect continuous attentional control or updating processes involved in complex tasks such as reading (e.g., Richmond et al., 2022; Burgoyne et al., 2025). Accordingly, the present findings should be interpreted as reflecting storage-based working memory processes. Although this approach is consistent with educational practice, it may not fully capture the complexity of attentional variability presentation. Future research incorporating task-based assessments may clarify the mechanisms underlying the observed associations. In addition, the cross-sectional design limits conclusions regarding developmental change. Longitudinal and experimental studies will be important. They can examine how attentional variability interacts with cognitive linguistic skills over time. They can also test whether the moderation pattern generalizes across contexts and languages (Vieiro-Iglesias & González-Fernández, 2025).

## 5. Conclusions

To our knowledge, although prior research has documented direct associations between inattention and reading outcomes, the possibility that inattentive symptom expression may alter the strength of relations between specific cognitive linguistic skills and comprehension has rarely been examined directly. The present findings extend this literature. They demonstrate that attentional variability is differentially related to the contribution of vocabulary knowledge in a nonreferred sample of fifth grade students. Reading comprehension therefore appears to reflect not only what students know. It also reflects how effectively they can sustain attention while applying that knowledge during reading.

## Figures and Tables

**Table 1:** Participants’ Description by Inattentive Symptom Expression Level (N = 267).

		Low Inattentive Symptom Expression (n = 183)		Elevated Inattentive Symptom Expression (n = 84)		Total		
		n	%	n	%	n	%	Χ^2^/t
Gender	Boys	78	42.6	52	61.9	130	48.7	8.123 **
	Girls	105	57.4	32	38.1	137	51.3	
School Type	State Secular	150	82.4	70	83.3	220	82.7	0.034
	State Religious	32	17.6	14	16.7	46	17.3	
Socioeconomic School Decile	1–3 (Strongest)	41	22.5	21	25.0	62	23.3	1.497
	4–7 (Average)	105	57.7	42	50.0	147	53.3	
	8–10 (Weakest)	36	19.8	21	25.0	57	21.4	
Age	M (SD)	10.81 (0.32)		10.82 (0.39)		10.82 (0.35)		−0.240

** *p* < 0.01.

**Table 2:** Descriptive Statistics and t-values for Word Reading Efficiency, Working Memory, and Vocabulary Size by Inattentive Symptom Expression.

Measure	Total (n = 267)	Low Inattentive Symptom Expression (n = 183)	Elevated Inattentive Symptom Expression (n = 84)	t (df)	*p*
Word Reading Efficiency	48.58 (13.13)	50.87 (12.30)	43.60 (13.57)	3.76 (145)	<.001
Vocabulary Size	23.37 (6.49)	24.26 (6.05)	21.44 (7.01)	4.19 (148)	<.001
Working Memory	3.04 (1.13)	3.15 (1.16)	2.79 (1.03)	2.52 (179)	.013

**Table 3:** Pearson Correlation Coefficients Between Reading Comprehension and Predictor Variables.

Variable	1	2	3
1. Reading Comprehension	-		
2. Word Reading Efficiency (without niqqud)	0.479 ***	-	
3. Vocabulary Size	0.524 ***	0.421 ***	-
4. Working Memory	0.251 ***	0.278 ***	0.206 ***

Note. *** *p* < .001 (2-tailed) for all correlations.

**Table 4:** Regression Coefficients for Predictors of Reading Comprehension.

			95% CI		
Predictor	β	SE	LL	UL	*p*
(Constant)		0.681	−1.497	1.184	.818
Word Reading Efficiency	0.301	0.010	0.032	0.070	<.001
Vocabulary Size	0.385	0.020	0.098	0.175	<.001
Working Memory	0.104	0.105	0.002	0.418	.047
Gender	0.054	0.231	−0.207	0.705	.283
Inattentive Symptom Expression	0.064	0.258	−0.188	0.827	.217

**Table 5:** Conditional effects of Vocabulary Knowledge on Reading Comprehension by Inattentive Symptom Expression Level.

Attentional Symptom Expression (Moderator)	Effect	SE	t	*p*	95% CI LL	95% CI UL
0 (Low Inattentive Symptom Expression)	0.2191	0.0239	9.1692	<.001	0.1721	0.2662
1 (Elevated Inattentive Symptom Expression)	0.1272	0.0305	4.1731	<.001	0.0672	0.1872

Note. Conditional effects (simple slopes) were estimated using PROCESS Model 1. Attentional symptom expression was derived from parent and teacher DSM-5-based rating scales and used as a dimensional indicator rather than a clinical diagnosis. SE = standard error; CI = confidence interval.

## Data Availability

The data presented in this study are available on request from the corresponding author due to ethical and privacy restrictions.
